# Random Lasers as Social Processes Simulators

**DOI:** 10.3390/e25121601

**Published:** 2023-11-29

**Authors:** Alexander Alodjants, Peter Zacharenko, Dmitry Tsarev, Anna Avdyushina, Mariya Nikitina, Andrey Khrennikov, Alexander Boukhanovsky

**Affiliations:** 1 Institute of Advansed Data Transfer Systems, ITMO University, 197101 St. Petersburg, Russia; alexander_ap@list.ru (A.A.); p.zaxarencko2015@yandex.ru (P.Z.); dmitriy_93@mail.ru (D.T.); avdushina98@gmail.com (A.A.); 79214406690@ya.ru (M.N.); 2 National Center for Cognitive Research, ITMO University, 197101 St. Petersburg, Russia; avb_mail@mail.ru; 3 International Center for Mathematical Modeling in Physics, Engineering, Economics, and Cognitive Science, Linnaeus University, S-35195 Vaxjo-Kalmar, Sweden

**Keywords:** quantum-like simulators, random lasers, superradiance, complex networks, decision-making agents, innovation diffusion

## Abstract

In this work, we suggest a quantum-like simulator concept to study social processes related to the solution of NP-hard problems. The simulator is based on the solaser model recently proposed by us in the framework of information cascade growth and echo chamber formation in social network communities. The simulator is connected with the random laser approach that we examine in the *A* and *D*-class (superradiant) laser limits. Novel network-enforced cooperativity parameters of decision-making agents, which may be measured as a result of the solaser simulation, are introduced and justified for social systems. The innovation diffusion in complex networks is discussed as one of the possible impacts of our proposal.

## 1. Introduction

Recently, growing interest has evoked the creation of quantum-inspired algorithms for solving NP-complete problems in economics, business, and finances [1]. Mathematically, they are related to the Karp’s 21 NP-complete problems [2]. As a rule, these problems are based on the QUBO (quadratic unconstrained binary optimization) procedure that requires finding a global minimum of some objective function [3]. At the physical level, such a function directly relates to the interaction Hamiltonian, onto which the QUBO mathematical problem defined on some graph can be mapped. In particular, for these purposes, various Ising Hamiltonians, which are described by the interaction of simple quantum-like two-level or 1/2-spin-like systems, are suitable [4].

Thus, it is possible to simulate some rather complex optimization processes in the field of economics, logistics, finances, etc. on a relatively simple physical (analog) information processing device that is currently called the (coherent) Ising machine (IM).

Physically, IMs represent gain-dissipative devices, c.f. [5]. From a technical point of view, IMs can be designed on the basis of photonic, electronic, and other platforms by using well-developed optoelectronic, semiconductor, and silicon technologies [6]. Notably, in many cases (quantum) spin properties may be realized by binary phases of optical signal processing, some properties of FPGA, CMOS, etc. [7]. In this regard, such devices may not have a quantum advantage, which is still an actual and yet unresolved practical problem. However, they are able to solve NP-hard (QUBO) problems much faster than conventional processors, since they can use well-established optical interference effects and possess vanishing overhead [8].

Moreover, various Ising machines are already commercially available; the main task for engineers and physicists is to increase the number of spins, *N*, for information processing, which is currently in the realm of tens of thousands or more, cf. [9]. Strictly speaking, at the same time, its operation raises questions that are not only practical but also fundamental.

One of the fundamental issues is the possibility of creating simulators, which may take into account decision-making (DM) processes occurring with the agents of natural (NIA) (humans) and/or artificial (AIA) intelligence cf. [10]. Notably, DM problems represent an important part of behavioral economics, management, and finance at a “microscopic” level approach to the problems, which remain in the focus of optimization tasks, cf. [11]. The “microscopic” level presumes the online information exchange between DM agents within some networks. However, the current mathematical formalization of optimization problems includes “tracing out” the information field itself, which plays a significant role in DM processes. This means that QUBO tasks are based on the efficient market hypothesis that deals with some rational DM agents’ activities, cf. [12,13]. In this framework, statistical (thermodynamically equilibrium) Ising models are widely exploited for the description and simulation of some (social) network features [14,15,16,17,18]. Therefore, Ising-like models presuming coupling between spin-like (effectively two-level) systems may be used to study collective opinion formation and social impact [19,20], for epidemic, information, and rumor spread in the network environment [21,22,23]. Nevertheless, such models are not enough to describe real-life social, cognitive information-driving processes occurring with DM agents in a network.

Studying DM problems for NIAs in the presence of their interaction with each other and with AIAs (chatbots, avatars, etc.) traditionally represents a hot topic in the applied mathematics, artificial intelligence, and control theory and may be resolved in the framework of agent-based modeling; see, e.g., [24,25,26]. However, the situation sometimes becomes more complex and diverse for real-life problems. Usually, agents communicate with each other in the framework of social, professional, and other networks, which can affect (rational) decision-making processes. Even in the case of simple rules (with binary preferences) of the DM agent behavior, the solution of a mathematical problem that accounts for the network environment (which introduces some uncertainties) becomes complicated and requires a certain interpretation, cf. [10].

In this regard, it makes sense to model DM agents by simple physical two-level or spin systems, whose behavior is ruled by the universal (in nature) quantum theory approach. More precisely, we are talking about socially oriented simulators, which can help in finding optimized *macroscopic* features of DM agents from their behavior at the *microscopic* level of interaction within various complex networks. Since agents can cooperate by exchanging information or receiving it from the outside, such simulators are dissipative-driven open systems; therefore, the information may be significantly weakened or amplified. To solve such an optimization problem, a physical simulator may be developed on the principles of interaction of the quantized field with a spin system [27].

Notably, the idea of describing self-organization processes in complex systems far from thermal equilibrium and inspired by laser theory was proposed a long time ago, cf. [28,29,30]. These works stimulated the development of the theory of social lasers (solaser) as a part of quantum-like modeling of biological, cognitive, social, and political processes [27,31,32,33,34]. Such modeling explores the mathematical formalism and methodology of quantum theory outside of physics without offering genuine quantum physical processes. Quantum-like systems are treated as information processors and the quantum information theory is in use [35,36,37]. However, the question of what the active medium that reproduces laser features beyond physics and simulates social activity is in the real world remains open.

Recently, in [38], we showed that a complex network may be recognized as a laser medium, containing simple two-level (spin-like) DM agents. In the framework of the social atom concept (see, e.g., [39]), these (mental) levels characterize two (“spin-up” and “spin-down”) mutually exclusive states of the agent in decision making. In the presence of information exchange and an external (information) strong pump field, DM agents absorb and emit so-called socially actual s-photons (which are short messages, e-mails, tweets, etc.), as happens with quantum two-level systems in conventional lasers. In fact, the spontaneous and stimulated behavior of DM agents removes the question of their rationality. As we showed in [38], simple rules based on the quantum theory approach can be attributed to DM agents; see, e.g., [37,40]. These rules can explain the information diffusion in networks as well as the growth in information cascades [41]. This approach to social phenomena description and determination matches the aforementioned solaser model due to its ability to enhance information spreading that occurs as a result of complex network peculiarities. A simplified quantum-like two-level (spin) DM agents model may be used in this case; DM agents represent so-called social atoms [39]. However, in [38], the problem of which parameters could be experimentally observed for the solaser remained unsolved. This problem is complex enough since it directly relates to measurements in large social systems.

In this work, we discuss the ways of solving the aforementioned problems. We propose a *social laser simulator* as a part of a more general decision-making supporting system. The measurement of the cooperativity parameter, which characterizes open quantum systems, and is well known in quantum physics [42], allows us to estimate the behavior and degree of cooperation of DM agents operating in the original social network environment.

Notice that the solaser concept is broad enough and may be applied to diverse problems occurring in social sciences, management, logistics, and finance, where the network environment plays a significant role in the agent’s decision-making problem. For example, we refer here to the innovation diffusion problem that considerably changed in recent decades due to the agents’ interaction and communication within social networks; see, e.g., [43].

The paper is arranged as follows. In Section 2, we represent the key features of networks and some important ideas on how we can create a solaser simulator. We examine regular and probabilistic networks obeying the power-law degree distribution (PLDD). In Section 2, we also discuss various types of lasers, which may be characterized by so-called cooperativity parameters of matter–field interaction and potentially could be interesting for the solaser simulator design. This section aims to analyze lasers, which exploit “bad” cavities or use some other physical effects for photon localization within the gain environment. In Section 3, we establish the model of a solaser simulator. We analyze this model in the framework of *A*- and *D*-class (superradiant) lasers relevant to the field diffusion, which exhibit field amplification in two different limits of spontaneous emission and dephasing rates. We specify the key features of superradiant laser characterized by the establishment of macroscopic polarization in the spin system due to the spin–spin correlations provided by the photonic field. In Section 4, we summarize our results establishing the key dependence for the normalized field amplitude, collective polarization, and population imbalance for the networks with two-level systems. We also discuss the application of our proposal for innovation diffusion specification and forecasting.

## 2. Materials and Methods

### 2.1. Network Architectures

In the literature, different approaches explain the real-world network topology [16,44]. Typically, real-world networks possess a large number of nodes, N≫1, that admit exploiting continuous degree distribution p(k), where *k* is the node degree variable. First, we examine regular networks when $p\left(k\right)=\delta \left(k-k_{0}\right)$, where $k_{0}$ is constant. Second, for our purposes, we use networks that obey the power-law degree distribution (PLDD) with p(k) established as

(1) $$\begin{array}{c} p\left(k\right)=\frac{\left(\eta -1\right)k_{min}^{\eta -1}}{k^{\eta }}, \end{array}$$

where η is the degree exponent; $k_{min}$ is the smallest node degree for which Equation (1) holds. p(k) obeys the normalization condition

(2) $$\begin{array}{c} \int_{k_{min}}^{+\infty }p(k)dk=1. \end{array}$$

Notably, such networks possess the preferential attachment phenomenon, which results in the hub’s appearance. The largest hub is described by degree $k_{max}$ called a natural cutoff. The condition

(3) $$\begin{array}{c} \int_{k_{max}}^{+\infty }p(k)dk=\frac{1}{N} \end{array}$$

can be used if the network with *N* nodes possesses more than one node with $k>k_{max}$. From (2) and (3), we immediately obtain $k_{max}=k_{min}N^{\frac{1}{\eta -1}}$.

The behavior of PLDD networks essentially depends on power degree η within allowed region 1<η<+∞, which covers the anomalous (1<η<2), scale-free (2<η<3), and random (η>3) regimes, cf. [45]. The properties of scale-free networks possessing distribution (1) for η=2 and η=3 should be calculated separately.

**Figure 1:**
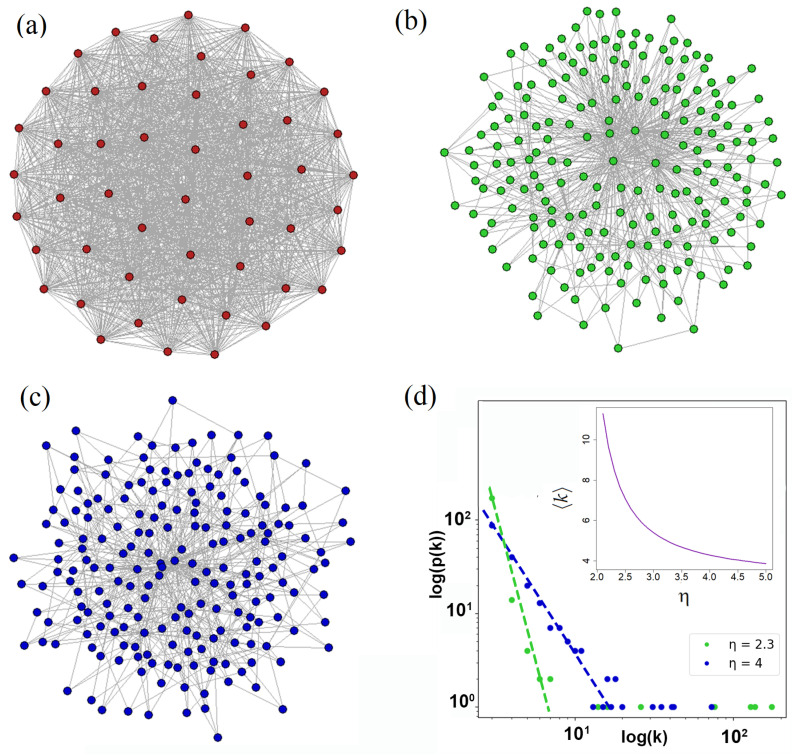
(**a**) Complete and PLDD graphs with N=50 and N=200 nodes, respectively, for (**b**) η=2.3 and (**c**) η=4. (**d**) PLDDs in a logarithmic scale for the networks given in (**b**,**c**). The dependence of 〈k〉 on power degree η is shown in the inset; $k_{min}=3$.

In Figure 1a–c, we plot networks under examination in this work. Figure 1a demonstrates a regular network with all-to-all node coupling. The real-world networks are mostly characterized by degree exponent η>2. In Figure 1b,c, we represent such networks with η=2.3 and η=4, respectively. The probability distribution function (1) plotted in a logarithm scale for these networks is shown in Figure 1d.

An important feature of PLDD networks is the existence of hubs, nodes with maximal node degree [45]. In Figure 1d, the hubs are clearly recognized by the set of dots located in the right corner of the distribution function p(k). The largest hub is described by degree $k_{max}$. Notably, the number of hubs and their size significantly grow when η→1.

Further, we exploit the average node degree parameter or simply the average degree,

(4) $$\begin{array}{c} \langle k\rangle =\int_{k_{min}}^{k_{max}}kp(k)dk, \end{array}$$

that determines the statistical properties of the network within the mean-field approximation. In particular, 〈k〉 indicates the number of connections on average available for an arbitrarily chosen node in the network. From (1)–(3), we can easily find the average degree 〈k〉 for the PLDD network within domains η>1, η≠2, and η≠3 in the form of

(5) $$\begin{array}{c} \langle k\rangle =k_{min}\frac{\eta -1}{2-\eta }\left(N^{\frac{2-\eta }{\eta -1}}-1\right), \end{array}$$

where N≫1 is the number of nodes. At η=2 and η=3, for average degree 〈k〉, we obtain $\langle k\rangle =k_{min}\ln N$ and $\langle k\rangle =2k_{min}$, respectively. As clearly seen from Figure 1d, 〈k〉 significantly increases with η→1. A regular network implies a constant number of connections between the nodes. It is maximal for the complete graph with *N* nodes and $\langle k\rangle =k_{0}=N-1$.

### 2.2. What Lasers Do We Need for the Solaser Simulator?

One of the interesting and intriguing problems that appear in the framework of solaser simulator design is how we can provide the cavity effect for social network simulation without an external cavity?

#### 2.2.1. Random Lasers

Current social networks serve versatile technical facilities in the information exchange and dissemination for DM agents and promote so-called echo chamber formation [46]. An echo chamber may be recognized as a rather new social phenomenon that occurs in a relatively close social network community, which provides selection and support of some contextual information preventing it from negation, other opinions, etc. The information localized within some part of the network circulates and reinforces primarily in a close community. As a result, the echo chamber effect in social communities evokes a social polarization of the DM agents who make their decisions in agreement with the information circulated in the echo chamber, cf. [47].

Clearly, the role of echo chambers in current networks is reminiscent of the role of a cavity that provides feedback in common lasers. Although current photonic technologies supply various cavities for photon trapping and localization (see, e.g., [48]), it may be challenging to develop a network structure using them. Thus, we need to obtain the cavity effect in the solaser simulator without an *external* cavity, if it is possible.

**Figure 2:**
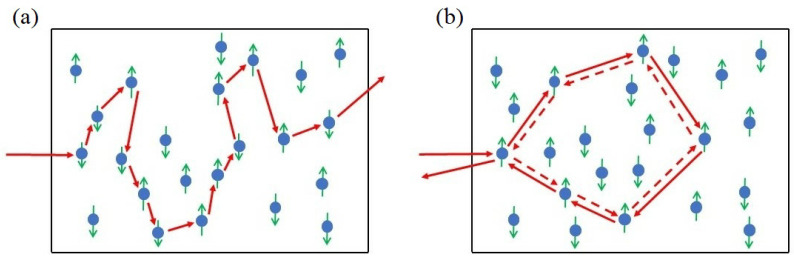
(**a**) Diffusive and (**b**) localized (Anderson) regimes of light scattering in random lasers that possess two-level (spin) systems. In random lasers, the angle between incident and outgoing light beams (not shown here) is small enough.

Fortunately, in modern photonics there exists a special class of lasers without external cavities at all; see, e.g., [49]. They are so-called random lasers where an electromagnetic (EM) field propagates through a disordered medium possessing the gain [50]. Technically, for random lasers, we can exploit a number of disordered materials, which provide light diffusion and amplification; see, e.g., [51]. In Figure 2, we represent a sketch of a random laser operating in two physically well-distinguished limits. The first regime in Figure 2a corresponds to the so-called diffusive limit of weak photon scattering when condition $\lambda <L_{ph}<L$ is fulfilled; λ is the wavelength, $L_{ph}$ is the mean free path of the photon in the medium, and *L* is its length. In this regime, the field spectrum exhibits inhomogeneous broadening that hides separate photonic modes.

The other, Anderson photon localization regime, is schematically shown in Figure 2b. It obeys a chain of inequalities $L_{ph}<\lambda <L$ and is relevant to the strong scattering regime with *coherent* feedback [49]. In this limit, photons after multiple scattering provide a closed-loop path that also includes a coherent (resonant) backscattering, which participates in constructive interference. Coherent lasing results from the combination of light amplification and interference along different loops. Intensity measurement demonstrates speckle patterns that establish the picture of various closed loops. Notably, although the chosen pattern forms accidentally average over a large number of realizations, we can obtain a quite clear picture of the processes of coherent laser field generation and amplification in the diffusive gain medium. In other words, we can recognize a random laser in the Anderson localization regime as a randomly distributed feedback laser where coherent (resonant) feedback is realized by diffusive elements.

Such a picture is quite close to the formation of echo chambers in the social network environment. Indeed, we can recognize Figure 2b in another way. It can be assumed that photons scatter along the paths that form random graphs. Recently, in [52], we offered a 2D material for the random laser that exhibits randomly formed networks with nodes that contain two-level systems (atoms, quantum dots, etc.). The network edges represent channels (waveguides) that provide coupling between the nodes. We studied low-threshold conditions for random lasing phenomenon, diffusion, and localization of radiation modes effects depending on the probability of couplings between arbitrary nodes. Thus, such a laser may be considered for the solaser simulator model.

#### 2.2.2. Superradiant Lasers

Another important effect that may be observed without any laser cavity is superradiance initially proposed by Dicke in his famous work [53]. In particular, Dicke showed that a collective of inverted two-level atoms can spontaneously transfer to the ground state within time $\tau_{c}$ inversely proportional to the number of atoms *N*, i.e., $\tau_{c}=\tau_{s}/N$, where $\tau_{s}$ is the spontaneous emission time in vacuum. As a result, the intensity of spontaneous emission of the atom ensemble is proportional to $N^{2}$. Physically, the superradiance phenomenon occurs due to the establishment of quantum correlations between the ensemble of TLSs or spins via collective interaction with the quantized EM field.

The practical realization of superradiance is to fulfill the following conditions. Initially, a short enough pump pulse incoherently creates the inversion in the two-level system, which then decays. Thus, the necessary conditions for the establishment of quantum correlations are

(6) $$\begin{array}{c} \tau_{c}\ll \tau_{s},T_{p}, \end{array}$$

where $T_{p}$ is the characteristic depolarization (dephasing) time for the TLS ensemble. In other words, condition (6) implies that collective processes should be much faster than individual relaxation processes, and we require a large number of atoms.

Up to now, superradiance has been extensively studied in theory and experiment; see, e.g., [54,55,56]. Recently, superradiance was demonstrated in trapped atoms [57], NV centers [58], quantum dots [59], etc. Currently, to study superradiance phase transition, investigations are used for the Dicke model under the condition of thermal equilibrium and small enough photon number, which may be recognized within the formal limit of $\tau_{s},T_{p}\to \infty$; see (6) and (12), [60]. In [61], we showed that the equilibrium superradiant phase transition may be obtained in complex network structures that we discuss in this work, cf. Figure 1b–d. We showed that the effective Rabi splitting may be enhanced $\sqrt{\langle k\rangle }$. In this regard, it seems to be important to establish a clear link between lasing and superradiant regimes in such a system beyond thermal equilibrium. Notably, in lasers, we can achieve the condition opposite to (12). We believe that it is useful to establish the physics of crossover from superradiance to laser. In this regard, one can assume that two-level systems are placed in the cavity as it occurs in the usual laser.

Recently, in [62], the authors demonstrated spontaneous synchronization of about $10^{6}$ atomic dipoles in the cavity that contains less than 0.2 photons on average. The steady-state superradiance was shown in the bad cavity limit, which implies a low lifetime (high optical losses) for intracavity photons. The practical realization of superradiance in this experiment is to fulfill a number of conditions. To elucidate such conditions, we introduce the *cooperativity parameter*

(7) $$\begin{array}{c} C_{0}\equiv \frac{g^{2}}{\kappa \gamma_{D}}, \end{array}$$

which usually characterizes the interaction of a single two-level system with a quantized field within various problems of quantum optics, cf. [42]. In (7), *g* is the single-photon Rabi frequency; κ, $\gamma_{D}≃1/\tau_{s}$ are the cavity decay and spontaneous emission rates, respectively.

Collective effects in the decay of the upper level occur with rate $\gamma_{c}≃C_{0}\gamma_{D}$. Thus, the superradiant state needs $\gamma_{P}$ much larger than $\gamma_{D}$ and depolarization rate Γ. More generally, the following inequality is to be fulfilled:

(8) $$\begin{array}{c} N\gamma_{c}\gg \gamma_{D}, \end{array}$$

which, in fact, represent some version of (6) at a steady state, cf. [63]. Notably, Equation (8) presumes a large value of the cooperativity parameter

(9) $$\begin{array}{c} C_{N}=NC_{0}=\frac{g_{N}^{2}}{\kappa \gamma_{D}}\gg 1, \end{array}$$

where $g_{N}\equiv g\sqrt{N}$ is the collective Rabi splitting parameter. In addition, it is instructive to introduce critical (intracavity) average photon ($N_{ph,c}$) and TLS ($N_{c}$) numbers (cf. [42])

(10) $$\begin{array}{c} N_{c}=\frac{\kappa \gamma_{D}}{g^{2}},\;N_{ph,c}=\frac{\gamma_{D}^{2}}{g^{2}} \end{array}$$

that specify critical quantum effects in collective matter–field interaction, which become important if conditions

(11) $$N_{c},\;N_{ph,c}\ll 1$$

are experimentally accessible. Equation (11) admits a simple interpretation: a single TLS and/or single photon may significantly alter the properties of the whole system. For common lasers in a classical regime, $N_{ph,c}\gg 1$, cf. [28].

Notably, for the superradiant state, the average number of photons $N_{ph}$ is smaller than the number of TLSs, i.e., condition

(12) $$\begin{array}{c} N_{ph}<N \end{array}$$

is fulfilled. From (10)–(12), one can find relation

(13) $$\begin{array}{c} \gamma_{D}\ll \kappa \end{array}$$

which implies the bad cavity limit when the photon decay rate κ is large enough.

In practice, common lasers as well as superradiant systems may be described in the framework of different laser classes. In this case, as we will see below, inequality (13) corresponds to the *D*-class lasers [64]; the opposite condition characterizes the *A*-class lasers. Thus, our main objective now is to show that both of these limits may be realized within some complex network structure due to the multimode regime of light–matter coupling; the transition from superradiant to usual, the *A*-class laser may be observed in this case.

## 3. Results

### 3.1. Mean-Field Equations for Solaser Simulators

**Figure 3:**
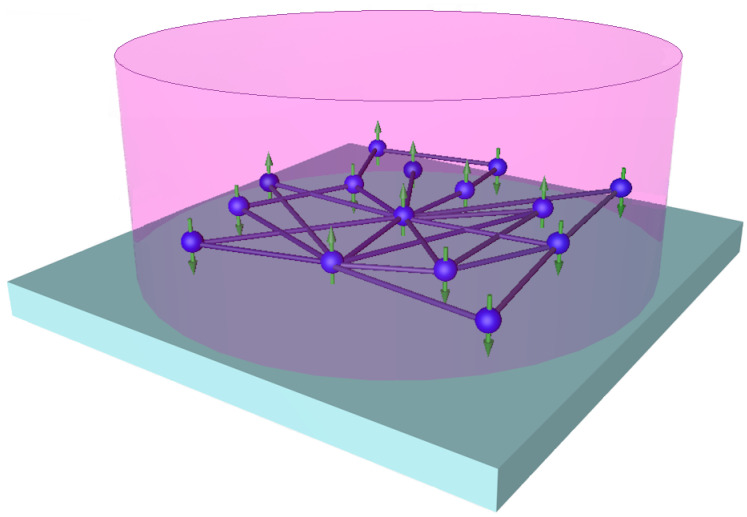
Sketch of the solaser simulator that represents an ensemble of two-level (spin) systems located within a complex network. In general, the network edges may represent projections of photon-guiding channels in the plane. All the system occupies an area of linear size L≤λ in the presence of a classical pump beam (pink cylinder).

We suppose that the solaser simulator may be represented as effectively 2D material that possesses a network interface shown in Figure 1. Strictly speaking, we can use 3D materials that provide required couplings between the nodes, cf. [65]. These couplings appear as network edges that represent projections onto the plane; see Figure 1. We also assume that TLSs have ${|g\rangle }_{i}$ (ground) and ${|e\rangle }_{i}$ (excited) states and occupy nodes of the complex network as shown in Figure 3. TLSs interact with quantized EM field by a set of waveguides, which represent edges of the graph and are described by annihilation (creation) $\hat{a_{j}}\left({\hat{a_{j}}}^{†}\right)$ operators. The control field, *R*, is a weak classical injection field (it is not shown in Figure 3). Practically, such a field may be realized by the interaction of TLSs with the classical (dressed) field, as it occurs in the framework of the dressed-state laser problem, cf. [66]. Thus, we represent the Hamiltonian of matter–field interaction in the form:

(14) $$\begin{array}{c} \hat{H}=\frac{1}{2}\sum_{i=1}^{N}\omega_{0,i}{\hat{\sigma }}_{i}^{z}+\omega_{ph}\sum_{i=1}^{M}{\hat{a}}_{i}^{†}{\hat{a}}_{i}+g\sum_{i=1}^{N}\sum_{j=1}^{\langle k\rangle }\left({\hat{a}}_{j}^{†}{\hat{\sigma }}_{i}^{-}+{\hat{a}}_{j}{\hat{\sigma }}_{i}^{+}\right)+iR\sum_{i=1}^{N}\left({\hat{a}}_{i}^{†}-{\hat{a}}_{i}\right), \end{array}$$

where ${\hat{\sigma }}_{i}^{z}={|e\rangle }_{ii}\langle e|-{|g\rangle }_{ii}\langle g|$ is the operator of the population inversion for *i*-th TLS (i=1,…,N); ${\hat{\sigma }}_{i}^{-}={|g\rangle }_{ii}\langle e|$, ${\hat{\sigma }}_{i}^{+}={\left({\hat{\sigma }}_{i}^{-}\right)}^{†}$ are the ladder operators; $\omega_{0,i}$ is the resonant frequency of the transition of *i*-th TLS from ground state ${|g\rangle }_{i}$ to excited one ${|e\rangle }_{i}$; *g* (Rabi frequency) represents the strength of interaction of a single TLS with a photonic mode of frequency $\omega_{ph}$. The number of modes is M≃N〈k〉, where 〈k〉 is the average node degree of the network structure. Hereafter, we use units with Planck constant ℏ=1.

Current quantum technologies provide a variety of facilities to realize 2D materials with network interface plotted in Figure 3. First, we can exploit two-level atoms trapped at the surface of a 2D structure by the Casimir–Polder effect leading to the attractive van der Waals forces, cf. [67]. Second, we can consider a photonic crystal membrane that possesses waveguides and contains a single layer of quantum dots arranged randomly at the network nodes [68]. Third, we can consider a network structure consisting of micropillars [69]. Each micropillar may be recognized as a TLS. Thus, we can broadly vary the material parameters of TLS networks depending on particular realization.

The operators defined in (14) obey usual commutation relations:

(15) $$\begin{array}{c} \begin{array}{c} \left[{\hat{a}}_{i},{\hat{a}}_{j}^{†}\right]=\delta_{ij}, \end{array} \end{array}$$

(16) $$\begin{array}{c} \begin{array}{c} \left[{\hat{\sigma }}_{i}^{z},{\hat{\sigma }}_{j}^{\pm }\right]=\pm 2\delta_{ij}{\hat{\sigma }}_{i}^{\pm }, \end{array} \end{array}$$

(17) $$\begin{array}{c} \begin{array}{c} \left[{\hat{\sigma }}_{i}^{+},{\hat{\sigma }}_{j}^{-}\right]=\delta_{ij}{\hat{\sigma }}_{i}^{z} \end{array} \end{array}$$

Without loss of generally, the system described by Hamiltonian (14) implies a set of Heisenberg equations:

(18) $$\begin{array}{c} \begin{array}{c} \frac{d\hat{E}}{dt}=-i\omega_{ph}\hat{E}-ig_{K}\sum_{i=1}^{N}{\hat{\sigma }}_{i}^{-}+\sqrt{\langle k\rangle }R, \end{array} \end{array}$$

(19) $$\begin{array}{c} \begin{array}{c} \frac{d{\hat{\sigma }}_{i}^{-}}{dt}=-i\omega_{0,i}{\hat{\sigma }}_{i}^{-}+ig_{K}{\hat{\sigma }}_{i}^{z}\hat{E}, \end{array} \end{array}$$

(20) $$\begin{array}{c} \begin{array}{c} \frac{d{\hat{\sigma }}_{i}^{z}}{dt}=2ig_{K}\left({\hat{E}}^{†}{\hat{\sigma }}_{i}^{-}-\hat{E}{\hat{\sigma }}_{i}^{+}\right), \end{array} \end{array}$$

(21) $$\begin{array}{c} \hat{E}=\frac{1}{\sqrt{\langle k\rangle }}\sum_{j=1}^{\langle k\rangle }\hat{a_{j}}, \end{array}$$

that obey bosonic commutation relation (15). In (18)–(20), parameter

(22) $$\begin{array}{c} g_{K}=g\sqrt{\langle k\rangle } \end{array}$$

characterizes the collective interaction of photonic field $\hat{E}$ with a single TLS. Equation (22) exhibits an important feature of the structure in Figure 1 and Figure 3: the collective (multimode) Rabi splitting may be enhanced $\sqrt{\langle k\rangle }$ times in comparison to the single mode case. We exploit (22) in (14) in the framework of the Gorini–Kossakowski–Sudarshan–Lindblad (GKSL) master equation that looks like (c.f. [70])

(23) $$\begin{array}{c} \frac{d\hat{\rho }}{dt}=-i\left[\hat{H},\hat{\rho }\right]+{\hat{L}}_{p}\left[\hat{\rho }\right]+{\hat{L}}_{D}\left[\hat{\rho }\right]+{\hat{L}}_{ph}\left[\hat{\rho }\right]+{\hat{L}}_{pump}\left[\hat{\rho }\right], \end{array}$$

where operators ${\hat{L}}_{j}$ characterize various decoherence processes and may be represented as

(24) $$\begin{array}{c} \begin{array}{c} {\hat{L}}_{ph}\left[\hat{\rho }\right]=\frac{\kappa }{2}\left(2\hat{E}\hat{\rho }{\hat{E}}^{†}-\hat{\rho }{\hat{E}}^{†}\hat{E}-{\hat{E}}^{†}\hat{E}\hat{\rho }\right), \end{array} \end{array}$$

(25) $$\begin{array}{c} \begin{array}{c} {\hat{L}}_{D}\left[\hat{\rho }\right]=\frac{\gamma_{D}}{2}\sum_{i}\left(2{\hat{\sigma }}_{i}^{-}\hat{\rho }{\hat{\sigma }}_{i}^{+}-{\hat{\sigma }}_{i}^{+}{\hat{\sigma }}_{i}^{-}\hat{\rho }-\hat{\rho }{\hat{\sigma }}_{i}^{+}{\hat{\sigma }}_{i}^{-}\right), \end{array} \end{array}$$

(26) $$\begin{array}{c} \begin{array}{c} {\hat{L}}_{p}\left[\hat{\rho }\right]=\frac{\Gamma_{2}}{2}\sum_{i}\left({\hat{\sigma }}_{i}^{z}\hat{\rho }{\hat{\sigma }}_{i}^{z}-\hat{\rho }\right), \end{array} \end{array}$$

(27) $$\begin{array}{c} \begin{array}{c} {\hat{L}}_{pump}\left[\hat{\rho }\right]=\frac{\gamma_{P}}{2}\sum_{i}\left(2{\hat{\sigma }}_{i}^{+}\hat{\rho }{\hat{\sigma }}_{i}^{-}-{\hat{\sigma }}_{i}^{-}{\hat{\sigma }}_{i}^{+}\hat{\rho }-\hat{\rho }{\hat{\sigma }}_{i}^{-}{\hat{\sigma }}_{i}^{+}\right), \end{array} \end{array}$$

where κ denotes the rate of photon losses in the structure in Figure 3; $\Gamma_{2}$ is the dephasing rate inherent to inhomogeneous broadening that occurs in the spectrum; $\gamma_{P}$ is the TLS incoherent pumping rate.

In this work, we propose a mean-field approach for Equations (21)–(27) solution that exploits factorization procedure for averages: $\langle {\hat{\sigma }}_{i}^{z}\hat{E}\rangle ≃\langle {\hat{\sigma }}_{i}^{z}\rangle \langle \hat{E}\rangle$, $\langle {\hat{\sigma }}_{i}^{+}\hat{E}\rangle ≃\langle {\hat{\sigma }}_{i}^{+}\rangle \langle \hat{E}\rangle$. Such an approach presumes neglecting quantum correlations between the field and TLSs. With the help of (14) and (21)–(27), we obtain

(28) $$\begin{array}{c} \begin{array}{c} \dot{E}=-\left(i\omega_{ph}+\frac{\kappa }{2}\right)E-ig_{K}NJ_{-}+\sqrt{\langle k\rangle }R, \end{array} \end{array}$$

(29) $$\begin{array}{c} \begin{array}{c} {\dot{J}}_{-}=-\left(i\omega_{0,i}+\frac{\Gamma }{2}\right)J_{-}+ig_{K}DE \end{array} \end{array}$$

(30) $$\begin{array}{c} \begin{array}{c} \dot{D}=\left(\gamma_{P}-\gamma_{D}\right)-\left(\gamma_{P}+\gamma_{D}\right)D+2ig_{K}\left(E^{*}J_{-}-EJ_{+}\right), \end{array} \end{array}$$

where dots denote derivatives with respect to time, and

(31) $$\begin{array}{c} \Gamma \equiv \gamma_{P}+\gamma_{D}+2\Gamma_{2}, \end{array}$$

is the total depolarization rate for the system shown in Figure 3. For further analysis, it is useful to introduce the cooperativity parameter

(32) $$\begin{array}{c} C_{0}\equiv \frac{g^{2}}{\kappa \Gamma }, \end{array}$$

which, in fact, represents generalization of Equations (7) for *A*- and *D*-class lasers analyzed below.

In (28)–(30), we introduce the collective mean-field variables:

(33) $$\begin{array}{c} E=\langle \hat{E}\rangle ,\;J_{\pm }=\frac{1}{N}\sum_{i=1}^{N}\langle {\hat{\sigma }}_{i}^{\pm }\rangle ,\;D=\frac{1}{N}\sum_{i=1}^{N}\langle {\hat{\sigma }}_{i}^{z}\rangle . \end{array}$$

Let us also transfer into (28)–(30) new variables by the rule $E\to Ee^{-it\omega_{0,i}}$, $J_{-}\to J_{-}e^{-it\omega_{0,i}}$ and $R\to Re^{-it\omega_{0,i}}$:

(34) $$\begin{array}{c} \begin{array}{c} \dot{E}=-\left(i\Delta_{i}+\frac{\kappa }{2}\right)E-ig_{K}NJ_{-}+\sqrt{\langle k\rangle }R, \end{array} \end{array}$$

(35) $$\begin{array}{c} \begin{array}{c} {\dot{J}}_{-}=-\frac{\Gamma }{2}J_{-}+ig_{K}DE, \end{array} \end{array}$$

(36) $$\begin{array}{c} \begin{array}{c} \dot{D}=\left(\gamma_{P}-\gamma_{D}\right)-\left(\gamma_{P}+\gamma_{D}\right)D+2ig_{K}\left(E^{*}J_{-}-EJ_{+}\right), \end{array} \end{array}$$

where $\Delta_{i}=\omega_{ph}-\omega_{0,i}$ is the detuning. Equations (34)–(36) are the subject of our analysis in this work.

### 3.2. *A*-Class Laser Simulator

Set of Equations (34)–(36) may be considered in the framework of various laser classes relevant to different ratios between key parameters of the system: κ, $\Gamma_{2}$, $\gamma_{D,P}$, and $\Delta_{i}$. For the *A*-class lasers, we suppose that the solution of (34)–(36) obeys condition

(37) $$\begin{array}{c} \Gamma ≃2\Gamma_{2}\gg \kappa ,g,\gamma_{D},\gamma_{P},\left|\Delta_{i}\right|. \end{array}$$

In particular, in (37), we assume that the depolarization rate is large enough due to dephasing parameter $\Gamma_{2}$; see (31). We eliminate polarization and population imbalance variables in this limit setting ${\dot{J}}_{-}=\dot{D}=0$. At the same time, we suppose field *E* to be a *real variable* assuming $\Delta_{i}=0$. From (34)–(36), we obtain

(38) $$\begin{array}{c} \begin{array}{c} \dot{E}=-\frac{\kappa }{2}E-ig_{K}NJ_{-}+\sqrt{\langle k\rangle }R, \end{array} \end{array}$$

(39) $$\begin{array}{c} \begin{array}{c} J_{-}=\frac{2ig_{K}}{\Gamma }DE, \end{array} \end{array}$$

(40) $$\begin{array}{c} \begin{array}{c} D=D_{0}+\frac{2ig_{K}}{\left(\gamma_{P}+\gamma_{D}\right)}\left(J_{-}-J_{+}\right)E, \end{array} \end{array}$$

where we introduce steady-state population imbalance

(41) $$\begin{array}{c} D_{0}=\frac{\gamma_{P}-\gamma_{D}}{\gamma_{P}+\gamma_{D}}. \end{array}$$

Solution of (34)–(36) and (38)–(40) for the *A*-class lasers may be performed in the framework of perturbation theory, cf. [28]. At the first-order perturbation level, we assume that the population imbalance is fixed, $D≃D_{0}$. Substituting it into (39) for polarization of the medium we obtain

(42) $$\begin{array}{c} J_{-}=\frac{2ig_{K}}{\Gamma }D_{0}E, \end{array}$$

Inserting (42) into (40), we can find the next approximation that characterizes corrections for the population imbalance:

(43) $$\begin{array}{c} D≃D_{0}\left(1-\frac{8g_{K}^{2}}{\Gamma (\gamma_{P}+\gamma_{D})}E^{2}\right). \end{array}$$

Next, inserting (43) into (39) and then into (38), we obtain

(44) $$\begin{array}{c} \dot{\Psi }=\left(\frac{2g_{K}^{2}ND_{0}}{\Gamma }-\frac{\kappa }{2}\right)\Psi -\frac{16g_{K}^{4}N^{2}D_{0}}{\Gamma^{2}\left(\gamma_{P}+\gamma_{D}\right)}\Psi^{3}+r, \end{array}$$

where we introduce normalized field amplitude

(45) $$\begin{array}{c} \Psi \equiv \frac{E}{\sqrt{N}}=\sqrt{\frac{N_{ph}}{N}}, \end{array}$$

that simply depends on the ratio of average photon number $N_{ph}$ and number of TLSs *N*. In (44), we also define effective control field parameter $r=\sqrt{\langle k\rangle }R/\sqrt{N}$ for the simplicity of notation.

Equation (44) is the mean-field equation characterizing field diffusion in the framework of (random) lasers with the network interface in the presence of control field *r* at r=0, relating to *supercritical pitchfork bifurcation* [71]. In our case, all network peculiarities are encoded in average node degree 〈k〉. The nonlinear term proportional to $\Psi^{3}$ plays an important role in the non-equilibrium phase transition occurring in the solaser. Therefore, for the steady-state solution of (44), $\dot{\Psi }=0$, we obtain

(46) $$\begin{array}{c} 0=A\Psi -B\Psi^{3}+r. \end{array}$$

In (46), we define

(47) $$\begin{array}{c} \begin{array}{c} A=\frac{2g_{K}^{2}N}{\Gamma }D_{0}-\frac{\kappa }{2}=\frac{\kappa }{2}\left(C_{\Gamma }D_{0}-1\right), \end{array} \end{array}$$

(48) $$\begin{array}{c} \begin{array}{c} B=\frac{16g_{K}^{4}N^{2}D_{0}}{\Gamma^{2}\left(\gamma_{P}+\gamma_{D}\right)}=\frac{C_{\Gamma }^{2}\kappa^{2}D_{0}}{\left(\gamma_{P}+\gamma_{D}\right)}, \end{array} \end{array}$$

where

(49) $$\begin{array}{c} C_{\Gamma }\equiv \frac{4g_{K}^{2}N}{\Gamma \kappa }=4C_{0}N\langle k\rangle \equiv 4C_{N}\langle k\rangle \end{array}$$

may be recognized as a total *network enforced cooperativity (NEC) parameter* that includes depolarization/dephasing rate Γ; $C_{N}\equiv C_{0}N$, cf. [42] and (7) and (9).

**Figure 4:**
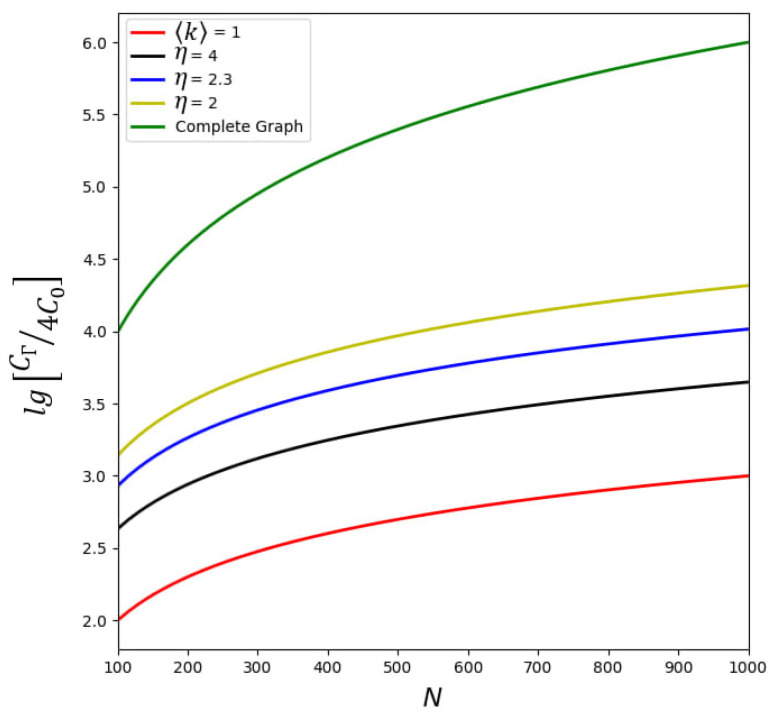
Dependence of the decimal logarithm of normalized NEC parameter $C_{\Gamma }/4C_{0}$ on the number of TLSs (equal to the number of nodes) *N* for the complete graph and various PLDD networks with $k_{min}=3$.

Figure 4 demonstrates the principal dependence of normalized NEC parameter $C_{\Gamma }/4C_{0}$ on particle number *N* inherent to the network system. For comparison, we represent the red line that corresponds to the case when the ensemble of TLSs interacts with a single-mode field; in this limit, we formally set 〈k〉=1 in (49). Significant enhancement of $C_{\Gamma }$ occurs for the PLDD network when power degree η decreases. It happens due to the enhancement of communication between TLSs. $C_{\Gamma }$ takes its largest value in the network that represents a complete graph; see the green line in Figure 4. In this limit, the NEC parameter $C_{\Gamma }\propto N^{2}$.

Equations (46)–(49) define the second order phase transition to lasing for real order parameter Ψ. The critical value of the parameters in (47)–(48) at r=0 obeys the equation

(50) $$\begin{array}{c} C_{\Gamma }D_{0}=1. \end{array}$$

Practically, we can choose population imbalance $D_{0,c}=1/C_{\Gamma }$ that implies the creation of initial inversion in the TLS network, or we can define the critical value of NEC parameter $C_{\Gamma ,c}$ that obeys Equation (50). In the latter case, we can choose the network parameter combination μ=〈k〉N as

(51) $$\begin{array}{c} \mu_{c}={\langle k\rangle }_{c}N_{c}=\frac{\Gamma \kappa }{4g^{2}D_{0}}. \end{array}$$

For the networks obeying condition $\mu \geq \mu_{c}$, the normalized laser field amplitude looks like

(52) $$\begin{array}{c} \Psi =\sqrt{\frac{A}{B}}\propto \Psi_{0}\sqrt{\frac{\mu }{\mu_{c}}-1}, \end{array}$$

where

(53) $$\begin{array}{c} \Psi_{0}=\sqrt{\frac{\gamma_{P}-\gamma_{D}}{2\kappa }} \end{array}$$

is the normalized field amplitude in the vicinity of phase transition point $\mu =\mu_{c}$. Equation (53) is a very simple interpretation: the order parameter amplitude is nonzero when pumping rate $\gamma_{P}$ exceeds spontaneous emission rate $\gamma_{D}$.

Notice that the critical value of the average node degree, ${\langle k\rangle }_{c}$, also depends on the number of nodes *N* for various types of networks. For the complete network, 〈k〉≃N and $\mu_{c}≃N_{c}^{2}$. In the vicinity of critical value $\mu_{c}$ for r≠0, from (46), we obtain

(54) $$\Psi ={\left(2r^{\prime }\Psi_{0}^{2}\right)}^{1/3},$$

where $r^{\prime }=r/\kappa$ is the dimensionless control field amplitude.

At large enough NEC parameter $C_{\Gamma }\gg 1$, coefficient *A* in (47) is essentially positive, and we can obtain significant amplification of macroscopic field within the network structure that is proportional to $C_{\Gamma }\kappa D_{0}/2\propto \langle k\rangle N$. In this limit, for the laser average photon number from (45) and (52), we obtain

(55) $$\begin{array}{c} N_{ph}=\frac{\left(\gamma_{P}+\gamma_{D}\right)\Gamma N_{ph,c}}{8\gamma_{D}^{2}\langle k\rangle }, \end{array}$$

where the photon number $N_{ph,c}$ is defined in (10). Thus, for the complex network structure obeying condition 〈k〉≫1 (see the inset in Figure 1d), it is possible to achieve the purely quantum limit determined in (11).

### 3.3. *D*-Class Superradiant Laser Simulator

Now, let us examine the superradiant state formation for the 2D system described in Figure 3. Although the superradiant state is relevant to quantum features of TLS polarizations (dipoles), some vital properties may be elucidated in the framework of Equations (34)–(36).

In this limit, we consider the bad cavity limit described by inequalities

(56) $$\begin{array}{c} \kappa \gg \Gamma ,g,\gamma_{D},\left|\Delta_{i}\right|. \end{array}$$

Condition (56) enables elimination of EM field amplitude and control field from (34)–(36) setting $\dot{E}=0$ and $\Delta_{i}=0$, R=0. In turn, a weak photon field provides spontaneous synchronization of the TLS dipoles, and from (34)–(36), we obtain

(57) $$\begin{array}{c} \begin{array}{c} E=-\frac{2i}{\kappa }g_{K}NJ_{-}, \end{array} \end{array}$$

(58) $$\begin{array}{c} \begin{array}{c} {\dot{J}}_{-}=-\frac{\Gamma }{2}J_{-}+ig_{K}DE, \end{array} \end{array}$$

(59) $$\begin{array}{c} \begin{array}{c} \dot{D}=\left(\gamma_{P}-\gamma_{D}\right)-\left(\gamma_{P}+\gamma_{D}\right)D+2ig_{K}\left(E^{*}J_{-}-EJ_{+}\right). \end{array} \end{array}$$

First, consider the steady-state supposing ${\dot{J}}_{-}=\dot{D}=0$ in (57)–(59). Strictly speaking, *E* is complex now, while $J_{-}$ is real. In this case, from Equations (57) and (58) for population imbalance *D*, we obtain $D=1/C_{\Gamma }$. Substituting it into (59) by using (57) and (58), we find the normalized field amplitude dependence:

(60) $$\begin{array}{c} \left|\Psi \right|=\sqrt{\frac{\gamma_{D}}{2\kappa }}\sqrt{\gamma -1-\left(\gamma +1\right)\left(\gamma +1+G\right)\frac{1}{C_{\gamma }}}, \end{array}$$

where we introduce dimensionless pumping ($\gamma \equiv \gamma_{P}/\gamma_{D}$) and dephasing ($G\equiv 2\Gamma_{2}/\gamma_{D}$) rates. In (60), we define another NEC parameter

(61) $$\begin{array}{c} C_{\gamma }\equiv \frac{4g_{K}^{2}N}{\kappa \gamma_{D}}, \end{array}$$

that characterizes cooperative effects for superradiance in a complex network structure, cf. (49).

From (60), it is clear that normalized field amplitude |Ψ| persists the superradiant steady-state under condition

(62) $$\begin{array}{c} \gamma -1-\left(\gamma +1\right)\left(\gamma +1+G\right)\frac{1}{C_{\gamma }}\geq 0. \end{array}$$

To obtain critical values of pumping rate relevant to the edges of domain (62), we set |Ψ|=0 in (62) and obtain

(63) $$\begin{array}{c} \gamma_{1,2}=-1+\frac{1}{2}\left(C_{\gamma }-G\right)\mp \frac{1}{2}\sqrt{{\left(C_{\gamma }-G\right)}^{2}-8C_{\gamma }}. \end{array}$$

Equation (63) specifies the domain of the pumping field

(64) $$\begin{array}{c} \gamma_{1}\leq \gamma \leq \gamma_{2}, \end{array}$$

where lasing is allowed.

In (63), it is clearly seen that, apart from the *A*-class lasing regime (see (50)), the pumping rate possesses two thresholds. Assume

(65) $$\begin{array}{c} C_{\gamma }\gg 1,G; \end{array}$$

in the limit of (65) from (63) we obtain

(66) $$\begin{array}{c} \begin{array}{c} \gamma_{1}≃1, \end{array} \end{array}$$

(67) $$\begin{array}{c} \begin{array}{c} \gamma_{2}≃C_{\gamma }. \end{array} \end{array}$$

The first threshold, $\gamma_{1}$, characterizes the phase transition to laser when the pumping field compensates for spontaneous emission. The second, much higher threshold, $\gamma_{2}$, corresponds to the establishment of correlations between the spins. In this limit, $\gamma_{P}\gg \gamma_{D}$, and from (67) and (56), we obtain

(68) $$\begin{array}{c} \left|\Psi \right|≃\sqrt{\frac{\gamma_{P}}{2\kappa }}=\sqrt{2}\frac{g_{K}\sqrt{N}}{\kappa }. \end{array}$$

Equation (68) implies the normalized field amplitude features in the presence of superradiance formation. Further, it is useful to analyze two limits of (68).

Traditionally, the superradiant state is associated with the limit, when the number of average photons is less than the number of TLSs [54]. This limit corresponds to condition

(69) $$\begin{array}{c} g\sqrt{\langle k\rangle N}<\frac{\kappa }{\sqrt{2}}. \end{array}$$

In the opposite case of |Ψ|>1 ($g\sqrt{\langle k\rangle N}>\kappa /\sqrt{2}$), we deal with the superradiant laser limit that implies a large photon number, as it takes place in a usual (*A*-class) laser. The crossover occurs at

(70) $$\begin{array}{c} g\sqrt{\langle k\rangle N}=\frac{\kappa }{\sqrt{2}}. \end{array}$$

Thus, the experimental verification of condition (69) for network systems (see Figure 3) represents a challenging and non-trivial task, in particular for complete networks 〈k〉≃N and Ψ∝gN.

Finally, let us examine the dynamical properties of superradiance within bad cavity limit (56) and condition (65), respectively. Supposing $\dot{D}=0$ in (57)–(59), one can derive (cf. (44))

(71) $$\begin{array}{c} {\dot{J}}_{-}=\frac{\gamma_{D}}{2}\left(C_{\gamma }-\gamma \right)J_{-}-\frac{\gamma_{D}C_{\gamma }^{2}}{\gamma }J_{-}^{3}. \end{array}$$

From Equation (71), it is clear that for the superradiant laser, it is more suitable to consider macroscopic polarization $J_{-}$ as the order parameter instead of field Ψ. At $C_{\gamma }<\gamma$ macroscopic polarization of TLSs coherently decay. Dynamical phase transition to the lasing occurs at $C_{\gamma }=\gamma$, cf. (66). For normalized pumping rate γ, which obeys inequality $C_{\gamma }>\gamma$, one can obtain a laser-like enhancement of collective polarization $J_{-}$.

## 4. Discussion

**Figure 5:**
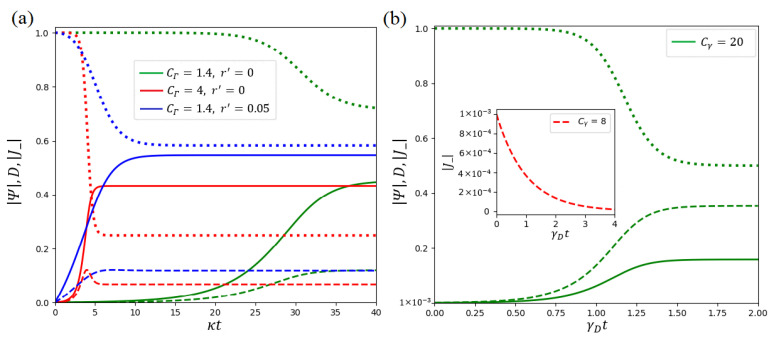
Mean-field dependence of normalized field amplitude |Ψ| (the solid lines), TLS polarization $|J_{-}|$ (the dashed lines), and population imbalance *D* (the dotted lines) on dimensionless time *t* for (**a**) *A*-class and (**b**) *D*-class (superradiant) lasers, respectively. The control field is normalized as $r^{\prime }\equiv r/\kappa$, $\Delta_{i}=0,D_{0}=1$. The other parameters are $\kappa /(\gamma_{P}+\gamma_{D})=0.5$; κ/Γ=0.1 for (**a**) and γ=10; $\gamma_{D}/\kappa =0.01$; $r^{\prime }=0$ for (**b**). Dependence of $|J_{-}|$ vs. $\gamma_{D}t$ below the threshold is shown in the insert to (**b**).

Let us summarize the results obtained. In Figure 5, we plot time dependent normalized field amplitude |Ψ|, collective polarization of TLSs $|J_{-}|$, and their population imbalance *D*, by solving Equations (44) and (71), respectively. The insert to Figure 5b clearly demonstrates collective decay of of polarization $|J_{-}|$ below the threshold $C_{\gamma }=\gamma$; see (71). All other curves in Figure 5 are plotted above the threshold values. As clearly seen, |Ψ| and $|J_{-}|$ demonstrate familiar S-shaped curve behavior. It is important that these curves are starting from small initial values. In particular, Figure 5b demonstrates small polarization $|J_{-}|=10^{-3}$ at t=0 that we use as a initial condition. Physically, such a polarization (and relevant photonic field) occurs in laser and/or superradiant systems due to quantum microscopic processes (quantum noise) that mean-field theory cannot fully describe. Simultaneously, population imbalance *D* (the dotted curves in Figure 5) decreases from its value $D_{0}=1$, which we can create via the pump field.

The curves in Figure 5a,b exhibit the important difference between *A*- and *D*-class lasers. TLS polarization $|J_{-}|$ follows normalized field amplitude |Ψ| and *D* behavior. On the contrary, in Figure 5b normalized field amplitude |Ψ| follows TLS polarization $|J_{-}|$. For *A*-class lasers, the dephasing rate of TLSs plays an essential role, while the spontaneous emission rate defines crucial properties of the superradiant laser. As a result, the dynamic processes in these two limits are determined by different cooperativity parameters (cf. (49) and (61)) and occur at various time scales shown on the abscissa axes in Figure 5a and Figure 5b, respectively.

Notably, all curves in Figure 5a grow faster if cooperativity parameter $C_{\Gamma }$ increases; see the red lines in Figure 5a. Macroscopic polarization possesses some maxima with a further increase in $C_{\Gamma }$. Such a behavior directly results from the cooperativity parameter enhancement due to the network peculiarities, cf. Figure 4. Similar behavior of TLS’s vital parameters occurs with the increasing of control field *r*; see the blue curves in Figure 5a. However, in this limit, the amplitude of the order parameter becomes much larger. Simultaneously, population imbalance may be negative, which indicates the lack of inversion. Notice that for the large control field our model is non-valid.

The curves in Figure 5 possess an important practical impact for socially mediated process studies. For example, we can consider the simulation of innovation diffusion, cf. [25]. In this case, the curves in Figure 5 may be connected with a number of consumers having adopted by time *t* and the bell curve of new adoptions. Notably, parameter estimation for diffusion models has represented a primary interest for a long time; see, e.g., [72]. It becomes especially crucial in the presence of a network environment [73]. Current theories and daily practice emphasize the importance of cooperative partnership and correlations of agents; see, e.g., [74,75]. In particular, our model of the simulator establishes a clear link between the *macroscopic* innovation diffusion model parameters and a number of the *microscopic* ones by DM agents’ NEC parameters $C_{\Gamma }$ or $C_{\gamma }$. Existing mathematical tools allow measuring an average node degree for a given social network estimating an average number of agents *N*; see, e.g., [45]. Thus, for a given innovation process, it is possible to infer information about one of the NEC parameters by the proposed random laser simulator. This circumstance makes it possible to comprehensively investigate social aspects of the innovation process and, perhaps, predict the likeliest scenarios of its development, depending on the microscopic parameter variation.

Notably, complex network peculiarities allow obtaining quantum regimes for the solaser simulator that we do not consider in this work due to the mean-field approximation that we exploit, cf. (55). In this sense, an intriguing problem occurs with the interpretation of the quantum limit for social systems possessing a large number of DM agents and cooperativity parameters (7), which obey conditions (11). Here, we suggest several hypotheses in analogy with quantum physics. We can recognize two possible realizations for the quantum limit of a social system. First, we can meet a limit when the average number of *s*-quanta (short messages, emails, etc.) is small enough and a few single messages can significantly manipulate the social system. Second, we can recognize the quantum limit for social systems as some limit when individual DM agents can have a significant influence on the behavior of other agents. Evidently, the proposed hypotheses may have a fairly broad interpretation in current social studies; their verification and refinement can open the way to new opportunities for research and forecasting the behavior of social communities affiliated with networks.

## Data Availability

All data used during this study are available within the article.

## Abbreviations

The following abbreviations are used in this manuscript:

AIA	Artificial intelligence agent
DM	Decision making
GKSL	Gorini–Kossakowski–Sudarshan–Lindblad
NEC	Network enforced cooperativity
NIA	Natural intelligence agent
PLDD	Power-law degree distribution
Solaser	Social laser
TLS	Two-level system
